# Vacuum-assisted thrombectomy of massive inferior vena cava thrombosis

**DOI:** 10.1016/j.jvsv.2025.102273

**Published:** 2025-06-04

**Authors:** Salvatore Silipigni, Alberto Stagno, Agatino Cacciola, Francesco Costa, Antonio Micari, Antonio Bottari

**Affiliations:** a Department of Biomedical and Dental Sciences and Morphological and Functional Imaging, University of Messina, A.O.U. Policlinico ‘G. Martino’, Interventional Radiology Unit, Messina; b Interventional Cardiology Unit HUVV, IBIMA Malaga, CIBERCV, Malaga; c Department of Biomedical and Dental Sciences and Morphological and Functional Imaging, University of Messina, A.O.U. Policlinico ‘G. Martino’, Interventional Cardiology Unit, Messina

Inferior vena cava (IVC) thrombosis represents a rare and potentially fatal condition affecting a minimal percentage of patients who develop deep vein thrombosis. In cases where anticoagulation fails, thrombolysis and thromboaspiration have gained a decisive role.^1^ Although non-thrombolytic mechanical thrombectomy devices have been profusely reviewed,^2^^,^^3^ to our knowledge, IVC thrombectomy managed with the Lightning System by Penumbra is still at its dawn.^1^^,^^4^

A 46-year-old male with medical history of deep vein thrombosis and pulmonary embolism treated with anticoagulation and IVC filter implantation 3 months earlier (Denali, Bard Medical), was admitted to the emergency room for sciatica-like pain and paresthesia and worsening weakness to lower limbs. Symptoms progressed to presyncope and arterial hypotension.

Venous computed tomography angiography (CTA) revealed massive thromboembolism of IVC and iliofemoral veins with mild pulmonary embolism.

After 24 hours of anticoagulation (Fondaparinux 7.5 mg/day) without clinical improvement, endovascular treatment was planned.

Bifemoral venography confirmed poor opacification and eccentric flow both in iliac veins and IVC with thrombosis extending across the filter mesh (*A/left*; Supplementary Video 1, online only).

Thromboaspiration was performed using Indigo Lightning 12F catheter and Separator 12 (Indigo Lightning, Penumbra) up to the filter meshes, carefully guiding the aspiration catheter over the Separator as a guidewire, caring not to migrate the filter (*A/middle*; Supplementary Video 2, online only).

Aspiration below the filter results were unsatisfactory (*A/right*; Supplementary Video 3, online only) due to floating material adherent to the filter; therefore, additional aspiration was performed through jugular access.

Careful thromboaspiration was performed above and across filter meshes with the 12F aspiration catheter (*B/left*; Supplementary Video 4, online only) until good flow was restored, allowing uncomplicated filter substitution (*B/right*; Supplementary Video 5, online only); a filter of the same type was used for substitution.

A significant amount of thrombus in different stages of organization was collected in the aspiration basket (*C/left*), whereas residual clot floating attached to the filter consisted mainly of material with high-fibrin content (*C/right*).

The patient reported relief of symptoms after the procedure and was able to stand the day after.

*D* (cover) compares admission venous CTA (*D/left*) and control CTA with near-complete thrombus removal, with few residual clots adherent to the caval wall (*D/right*).

Compared with larger bore thromboaspiration systems,^1^^,^^5^ the Lightning 12 System proved to be effective and safe. Potential technical limitations are overcome with the introduction of the 16Fr Lightning Flash 2.0 System, which features dual clot detection algorithms, resulting in more powerful aspiration and reduced blood aspiration.^4^

**Figure:**
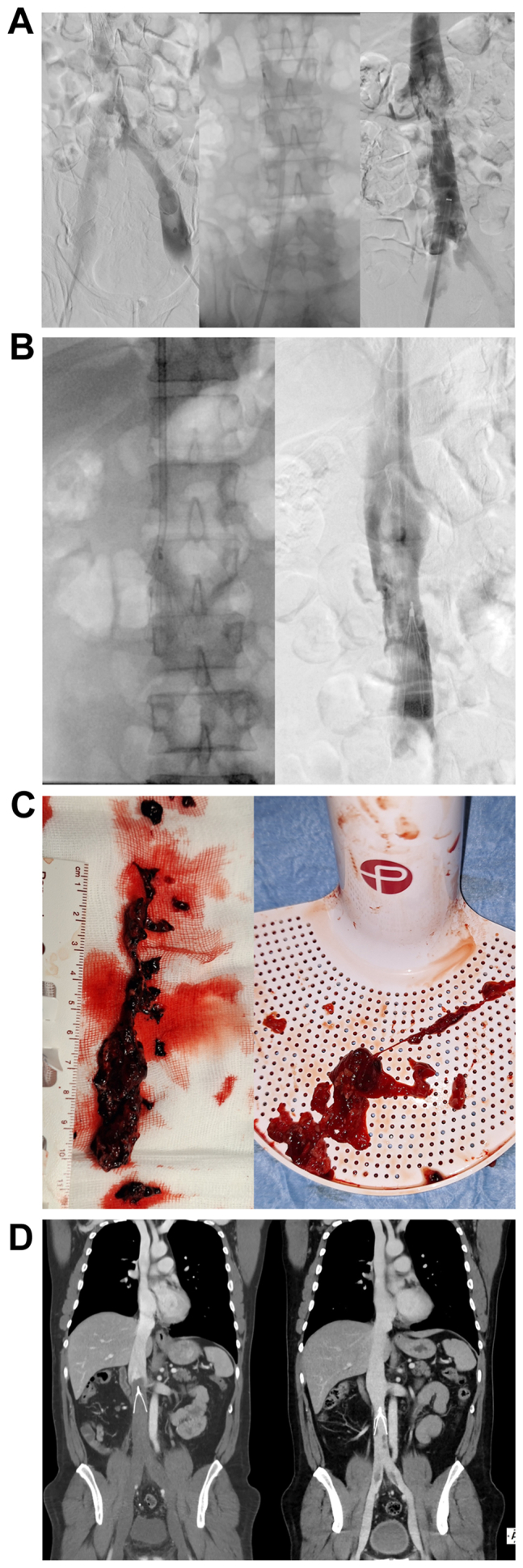

The patient signed informed consent for publication.

## Funding

None.

## Disclosures

None.

## Figures and Tables

**Cover image:**
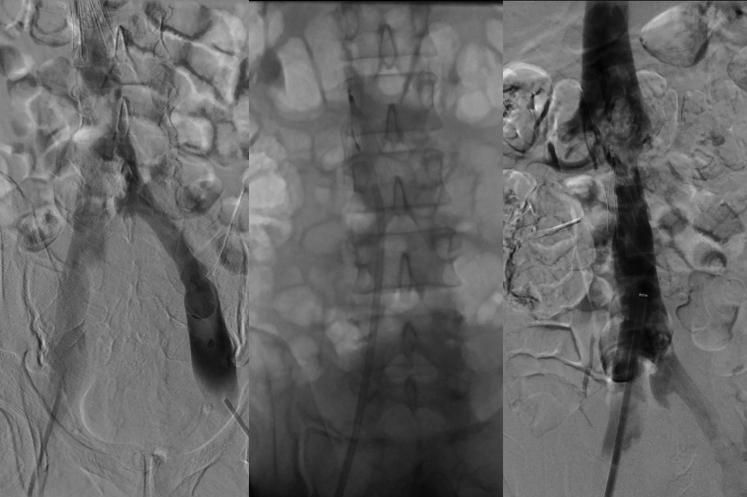


## Appendix (online only)

Supplementary Video 1 (online only) Venography performed via simultaneous bilateral femoral sheath injections demonstrates delayed, eccentric flow with poor opacification of the iliac veins and inferior vena cava (IVC).

Supplementary Video 2 (online only) Thromboaspiration performed via right femoral access using an Indigo Lightning 12F catheter. The Separator 12 device was employed not only to safely fragment the thrombus but also to guide the aspiration catheter through the filter meshes with controlled advancement, thereby minimizing the risk of cranial filter migration.

Supplementary Video 3 (online only) Venography through the 12F introducer sheath reveals persistent floating thrombotic material adherent to the right wall of the IVC and within the filter. Further aspiration via the femoral route was considered hazardous; therefore, jugular access was selected to continue the procedure.

Supplementary Video 4 (online only) The procedure was continued via jugular access. Aspiration was performed using the Indigo Lightning 12F catheter both above and through the filter meshes with good control. While aspiration through the filter was unremarkable, attempts to aspirate along the right IVC wall were limited by severe pain reported by the patient. Aspiration was discontinued upon restoration of adequate IVC flow. Approximately 470 mL of blood were collected in the aspiration canister at the end of the procedure.

Supplementary Video 5 (online only) Venography following filter replacement. After thromboaspiration, the filter was replaced with a new device of the same type (Denali, Bard Medical), positioned at the level of the third lumbar vertebral body. A residual thrombus remains adherent to the right IVC wall. Despite this, brisk flow was restored, achieving a satisfactory hemodynamic result.
